# The Enhancing Effect of Fungal Immunomodulatory Protein-Volvariella Volvacea (FIP-vvo) on Maturation and Function of Mouse Dendritic Cells

**DOI:** 10.3390/life11060471

**Published:** 2021-05-24

**Authors:** Ju-Pi Li, Yi-Pang Lee, Jung-Chein Ma, Betty-Revon Liu, Nien-Tsu Hsieh, Dz-Chi Chen, Ching-Liang Chu, Ren-In You

**Affiliations:** 1 Department of Pathology, School of Medicine, Chung Shan Medical University, Taichung 40201, Taiwan; d888203@gmail.com; 2 Department of Pediatrics, Chung Shan Medical University Hospital, Taichung 40201, Taiwan; 3 Department of Health Administration, Tzu Chi University of Science and Technology, Hualien 97004, Taiwan; candybonbon@tzuchi.com.tw; 4 Division of Oral Pathology, Department of Dentistry, Tzu Chi General Hospital, Hualien 97004, Taiwan; 5 Graduate Institute of Immunology, College of Medicine, National Taiwan University, Taipei 10051, Taiwan; tma.eternal@gmail.com; 6 Department of Laboratory Medicine and Biotechnology, Tzu Chi University, Hualien 97004, Taiwan; brliu7447@gms.tcu.edu.tw; 7 Yeastern Biotech Co., Ltd., New Taipei City 22180, Taiwan; hsiehnt@yb-biotech.com (N.-T.H.); jody.yeastern@gmail.com (D.-C.C.)

**Keywords:** fungal immunomodulatory proteins (FIPs), FIP-vvo, dendritic cell (DC)

## Abstract

Volvariella volvacea, also known as straw mushroom, is a common edible mushroom in Chinese cuisine. It contains many nutrients for human health. A fungal immunomodulatory protein (FIP) has been isolated from *V. volvacea* and named FIP-vvo. Although the regulatory effects of many FIPs on immunity have been identified, the impact of FIP-vvo in modulating dendritic cells (DCs), which play a key role to connect the innate and the adaptive immunity, is not known. In this study, we aim to study the effect of FIP-vvo on the DC maturation and function. We found that FIP-vvo slightly increased the generation of CD11c^+^ bone marrow-derived DC (BMDC). In addition, the surface expression of MHCII was promoted in BMDCs after the treatment of FIP-vvo, suggesting that FIP-vvo induces DC maturation. Furthermore, FIP-vvo enhanced the ability of BMDCs to activate antigen-specific T cell responses in vitro. In the in vivo study, the FIP-vvo treatment facilitated T cell response in lymph nodes. Therefore, for the first time, our data demonstrated that FIP-vvo promoted DC maturation and function and suggested that FIP-vvo could have benefits for human health by enhancing immunity.

## 1. Introduction

Mushrooms comprise a number of edible fungi and have been included in meals daily around the globe due to their high nutritional and functional properties [1]. *Volvariella volvacea*, also known as paddy straw mushroom or straw mushroom, is commonly used in Chinese cuisine [2]. It belongs to the saprophytic fungus in the Pluteaceae family and Basidiomycota phylum with high nutritional components and unique taste, and there are plenty in the tropical and subtropical regions of Southeast Asia. In addition, *V.* *volvacea* is an important source for healthy foods and medicines to regulate immune responses. For example, some types of lectin isolated from *V. volvacea* can potentially stimulate lymphocyte proliferation via the increasing calcium influx [3]. Interestingly, the protein fractions from *V.* *volvacea* have been reported to promote cytokine production by peripheral blood mononuclear cells [3,4]. However, what protein component is contributing to the immune function remains to be identified.

A group of proteins have been isolated from various fungi with conservative structures, and they form a family named fungal immunomodulatory proteins (FIPs). FIPs can be divided into five subgroups: (1) Fve-type FIPs, which have the Fve family (*Flammulina velutipes*) domain signature (Pfam: PF09259); (2) Cerato-type FIPs, which contain the cerato-platanin family (*Ascomycete Ceratocystis platani*) domain signature (Pfam: PF07249); (3) PCP-like FIPs, which are classified by protein identity (*Poria cocos immunomodulatory protein*, PCP); (4) TFP-like FIP, which only contains *Tremella fuciformis protein* (TFP); and (5) unclassified FIP [5]. In 1997, Hsu et al. purified a protein from *V. volvacea* known as FIP-vvo [6]. Based on the sequence and structure, FIP-vvo belongs to the Fve-type FIPs. FIPs have various activities that affect immunity by regulating the cell-mediated or/and humoral immune system, resulting in anti-allergic activity [6,7,8,9], anti-inflammatory functions [10,11,12,13], and anti-tumor effects [14,15,16,17]. However, how FIP-vvo regulates the immune responses has not yet been studied.

Dendritic cells (DCs) are professional antigen presenting cells (APCs), which play a critical role in linking innate and adaptive immune responses [18]. Upon contact with pathogens, immature DCs phagocytose antigens and process antigens loading on major histocompatibility complexes (MHCs) molecules. At the same time, DCs become mature and migrate to secondary lymphoid organs [19]. The mature DCs produce pro-inflammatory cytokines, which help to determine the differentiation of specific types of T cells [20,21,22]. Because DCs are the key regulator in initiating immune responses, DCs have been developed as potent vaccines for the treatment of cancer and inflammatory diseases [18,23,24,25,26]. Thus, to discover the compounds that can modulate the DC function is an important strategy in biotechnology [27]. Natural or artificial substances that promote DC activation can potentially be candidates and applied to immunotherapy and vaccination [24].

Although the immunoregulatory functions of many FIPs have been reported, the functional role of FIP-vvo in immune responses is largely unknown. In this study, we investigated the effect of FIP-vvo on DC activation and function. We found that FIP-vvo promotes DC maturation and then enhances T cell activation. Our study may be helpful for the development of FIP-vvo as functional supplements for human health.

## 2. Materials and Methods

### 2.1. Mice

C57BL/6 mice were purchased from the National Laboratory Animal Center (Taipei, Taiwan). OT-II transgenic mice were provided by Dr. Clifford Lowell (UCSF, San Francisco, CA, USA). All mice were housed in the laboratory animal center at TCU, Taiwan, under an Institutional Animal Care and Use Committee-approved protocol: 108-11 (approved date: 27 December 2019). The C57BL/6 mice (6−8 weeks old) were bred under the specific pathogen-free condition. A maximum of 4 mice were kept in a cage, supplied with sterilized chow diet and water, and maintained at 23 °C with 12/12 h of light–dark cycle.

### 2.2. Preparation of DCs

Mouse DCs were generated from bone marrow (BM) as previously described [14,28]. Briefly, BM cells were isolated from the femur and tibia of the mice. After removing red blood cells, the cells were seeded into a 24-well plate using complete RPMI 1640 medium containing 2 mM L-glutamine, 1× non-essential amino acids (Simply^TM^), 2 mM sodium pyruvate (Simply^TM^), 2.4 mM HEPES (Simply^TM^), 0.1 mM 2-ME (Sigma), 1×penicillin/streptomycin (Simply^TM^), 10% FBS (Gibco), and 10 ng/mL recombinant mouse GM-CSF (PeproTech). On day 6, DC (>70%) was collected and used for all of the experiments.

### 2.3. Recombinant FIP-vvo Preparation

Recombinant FIP-vvo was provided by Yeastern Biotech Co., Ltd. (Taipei, Taiwan). Briefly, DNA sequence encoding FIP-vvo was cloned and expressed in *Saccharomyces cerevisiae*. Cells expressing FIP-vvo were disrupted and centrifuged, and the supernatant was passed through molecular sieves to obtain proteins between 10 kDa and 100 kDa. The filtrate was further purified using FPLC with Superdex 75 columns (GE Healthcare, Chicago, IL, USA), and then the purity was determined by FPLC using the GE AKTA purifier FPLC system (GE Healthcare, Chicago, IL, USA).

### 2.4. Apoptosis Assay

Apoptosis was determined by the Annexin V-FITC apoptosis detection kit (eBioscience, San Diego, CA, USA) according to the manufacturer’s protocol. In brief, BMDCs (1 × 10^6^/mL) were treated with medium (control) or 1–20 μg/mL FIP-vvo for 24 h. After treatment, DCs were washed twice with cold PBS and collected by centrifugation. The pellet was resuspended in binding buffer and stained with FITC-labeled Annexin V for 15 min at 4 °C in the dark. Then, propidium iodide was added for 5 min at 4 °C in the dark. The percentage of Annexin V-positive cells was determined with the BD FACSCanto II (BD Biosciences, San Jose, CA, USA) and analyzed with FlowJo analytical software (TreeStar, Woodburn, OR, USA).

### 2.5. DCs Maturation Analysis

BMDCs (1 × 10^6^/mL) were treated with medium or FIP-vvo (10 or 20 μg/mL) for 16 h. The phenotype of mature DCs was analyzed by staining with fluorescence-conjugated anti-CD11c, -CD80, -CD86, and -MHCII antibodies (BioLegend, San Diego, CA, USA). Then, the expression of mean fluorescence intensity (MFI) was determined by flow cytometry [29].

### 2.6. Measurement of Cytokine Assay

For intracellular cytokine staining, BMDCs (1 × 10^6^/mL) were stimulated with medium or FIP-vvo for 6 h and treated with a protein transport inhibitor containing brefeldin A, GolgiPlug (BD Bioscience) for 4 h before harvest. Cells were blocked with Fc receptor blocker and resuspended with fixation/permeabilization buffer (eBioscience) for 20 min at 4 °C. The intracellular cytokine was detected with fluorescence-conjugated anti-TNF-α, -IL-10, and -IFN-γ antibodies (BioLegend) and analyzed by flow cytometry.

For enzyme-linked immunoassay (ELISA), the supernatants were collected from BMDC cultures after medium or FIP-vvo treatment for 24 h. The production of TNF-α, IL-2, IL-12p70, and IL-6 was measured by ELISA kits (eBioscience) according to the manufacturer’s instructions. Briefly, anti-TNF-α, -IL-2, -IL-12p70, and -IL-6 antibodies were precoated in a 96-well plate using carbonate buffer. After incubating with the culture supernatants, detecting antibodies were added and then detected by adding HRP substrate. The OD values were determined by the ELISA reader.

### 2.7. Analysis of T cell Activation

#### 2.7.1. The In Vitro Assay

FIP-vvo-stimulated DCs were added with or without ovalbumin (OVA) peptide (2 μg/mL) for 3 h. At the same time, CD4-positive T cells were harvested from OT-II transgenic mice using a CD4 T cell isolation kit (STEMCELL Technologies) according to the manufacturer’s instructions. Then, T cells (1 × 10^7^/mL) were labeled using the CFSE Cell Division Tracker Kit (BioLegend) and cocultured with DCs at DC: T cell= 1:10. The percentages of the decreased CFSE levels were analyzed by flow cytometry [30,31].

#### 2.7.2. The In Vivo Assay

The enhancing effect of FIP-vvo on DCs for T cell activation in vivo was determined as described previously [31]. Randomized C57BL/6 mice were immunized with OVA mixed with incomplete Freund’s adjuvant (IFA) alone or IFA and FIP-vvo (10 μg) via footpad injection, and 3 mice were used for each group. After 10 days, total cells were harvested from draining lymph nodes and cultured with OVA for 3 days. T cell populations were analyzed by staining with fluorescence-conjugated anti-CD3, -CD4, and -CD8 antibodies (BioLegend) and flow cytometry. 

### 2.8. Statistical Analysis

All statistical analyses were performed using GraphPad Prism 8.3.0 (GraphPad Software Inc., La Jolla, CA, USA). The Mann–Whitney U nonparametric test was performed to evaluate the significant differences between these groups. The results were expressed as mean ± SD (standard deviation). All experiments were performed at least three times. Two-sided *p*-values less than 0.05 were considered statistically significant.

## 3. Results

### 3.1. FIP-vvo Promotes Maturation of DCs

To study the role of FIP-vvo in the immunomodulatory function of DCs, the cytotoxicity effect of FIP-vvo in DCs was first evaluated. DCs were treated with various concentrations of FIP-vvo for 24 h, and then the apoptosis was examined using Annexin V and propidium iodide double-staining analysis. 

**Figure 1:**
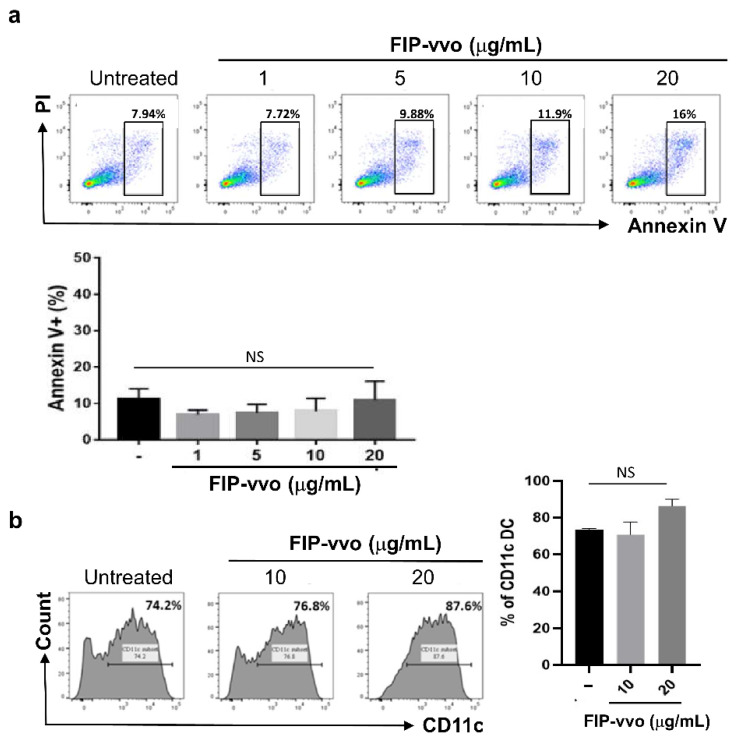
FIP-vvo does not induce cytotoxicity and DCs maturation. (**a**) DCs are treated with medium (untreated/-) or FIP-vvo (1−20 μg/mL) for 24 h. Cell death levels of DCs are analyzed by Annexin V and PI double staining. The percentage of Annexin V-positive cells is determined by flow cytometry (mean ± SD; NS, no significance; *p* > 0.05; FIP-vvo vs. untreated; *t*-test; *n* = 4/group). (**b**) DCs are treated with medium (untreated/-) or FIP-vvo (10 and 20 μg/mL) for 24 h. The expression levels of CD11c are determined by flow cytometry. Representative data and quantification are shown (mean ± SD; NS, no significance; FIP-vvo vs. untreated; *t*-test; *n* = 4/group).

As shown in Figure 1a, DCs showed 7.94% apoptosis in the normal growth condition. After 10 and 20 μg/mL FIP-vvo treatment, the DCs showed approximately 11.9 and 16% apoptosis, respectively (Figure 1a). Therefore, FIP-vvo (1−20 μg/mL) did not significantly induce the apoptosis level in DCs (Figure 1a). In order to elucidate the effect of FIP-vvo on DC differentiation in vitro, the CD11c levels of immature DCs (iDCs) were examined. The percentage of the CD11c-positive population slightly but not significantly increased in DCs treated with FIP-vvo compared to control cells (Figure 1b).

**Figure 2:**
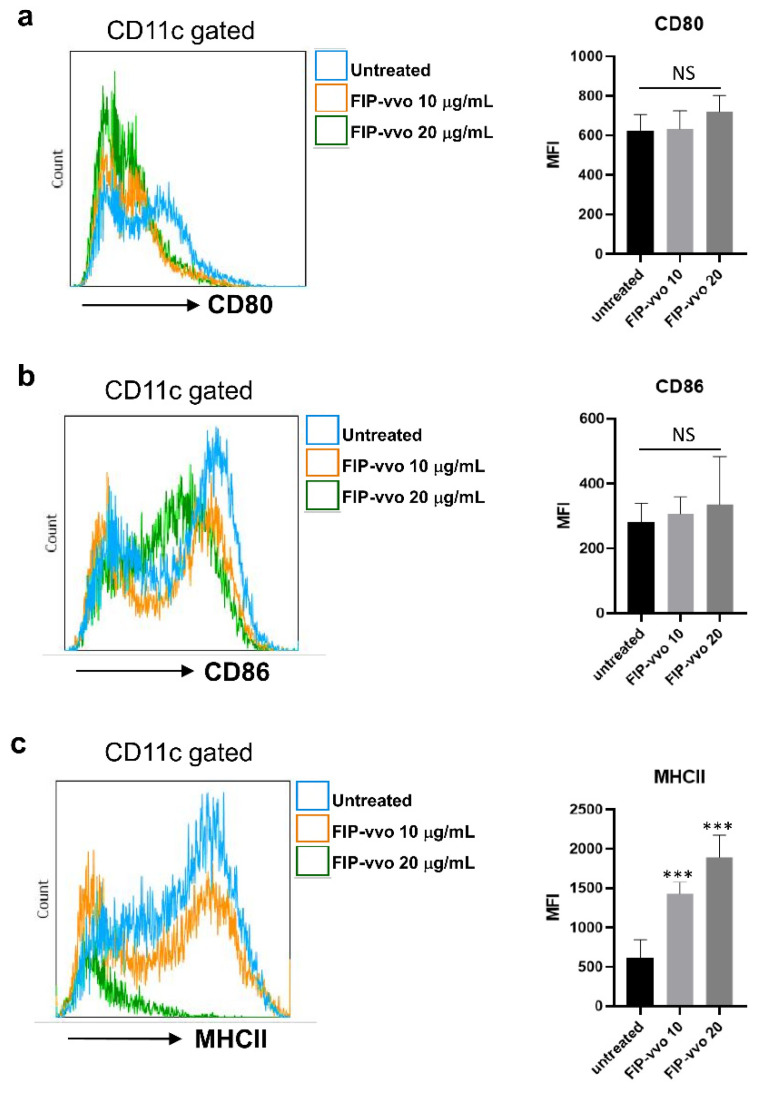
FIP-vvo induces the MHCII levels of DCs. DCs are treated with medium (untreated/-) or FIP-vvo (10 and 20 μg/mL) for 24 h. The mean fluorescence intensity (MFI) levels of CD80 (**a**), CD86 (**b**), and MHCII (**c**) are determined by flow cytometry. Representative data and quantification are shown (mean ± SD; NS, no significance; *** *p* < 0.005; FIP-vvo vs. untreated; *t*-test; *n* = 3/group).

After the iDCs recognized the microorganisms, they were activated by innate receptors and expressed the costimulatory molecules, CD80 and CD86, and simultaneously presented the antigen through MHCII to activate CD4-positive T cells [21]. Thus, the expression levels of CD80, CD86, and MHCII were examined in CD11c-positive DCs. As shown in Figure 2, FIP-vvo (10 and 20 μg/mL) treatment for 24 h significantly induced the MHCII level, but not CD80 and CD86, compared to the untreated group. The data suggest that FIP-vvo promotes maturation by enhancing the formation of MHCII for Ag presentation.

### 3.2. FIP-vvo Enhances the Inflammatory Cytokine Production by DCs

**Figure 3:**
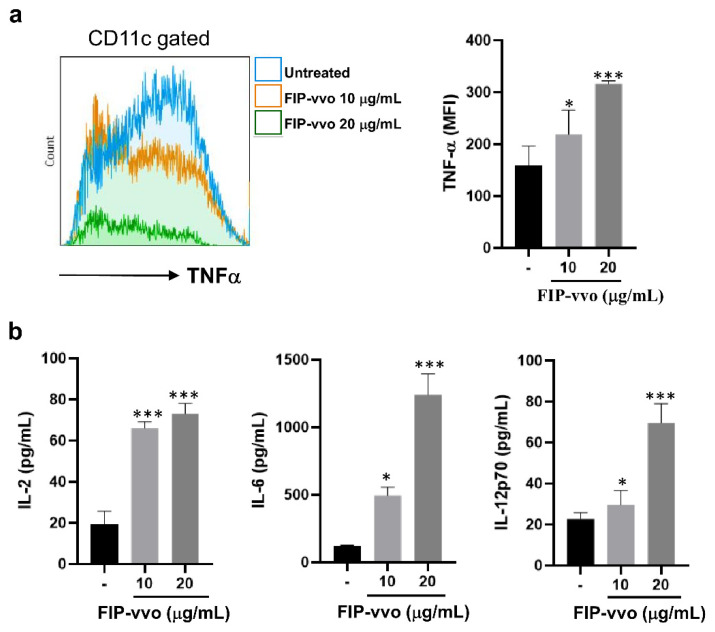
FIP-vvo induces TNF-α, IL-2, IL-6, and IL-12p70 levels of DCs. (**a**) DCs are treated with medium (untreated/-) or FIP-vvo (10 and 20 μg/mL) for 6 h and then added GolgiPlug, a protein transport inhibitor containing brefeldin A, for another 4 h. The intracellular TNF-α mean fluorescence intensity (MFI) levels are determined by flow cytometry. Data are gated on CD11c^+^ DC cells. The gray-filled area represents staining with an isotype-matched control antibody. (**b**) DCs are treated with medium (-) or FIP-vvo (10 and 20 μg/mL) for 24 h, and the culture supernatants are collected. The IL-2, IL-6, and IL-12p70 levels are determined by ELISA. Representative data and quantification are shown (mean ± SD; NS, no significance; * *p* < 0.05; *** *p* < 0.005; FIP-vvo vs. medium; *t*-test; *n* = 3/group).

DCs undergo a complex maturation process after capturing antigens and release a series of pro-inflammatory cytokines to regulate T cell responses [32]. To examine the effect of FIP-vvo on the pro-inflammatory cytokine secreted by DCs, two strategies were used. First, DCs were treated with FIP-vvo for 6 h, and then the intracellular TNF-α-producing cells were examined by flow cytometry. The FIP-vvo-treated DCs had a higher level of intracellular TNF-α compared to the untreated DCs (Figure 3a). 

Next, DCs were treated with FIP-vvo for 24 h, and then the supernatants were collected. The amounts of IL-2, IL-6, and IL-12p70 in the supernatants were determined by ELISA. The secreted IL-2, IL-6, and IL-12p70 levels were significantly enhanced in FIP-vvo-induced DCs (Figure 3b). These results indicate that FIP-vvo could activate the DCs to produce cytokines for regulating immune responses.

### 3.3. FIP-vvo Increases DC-Induced T cell Activation In Vitro

**Figure 4:**
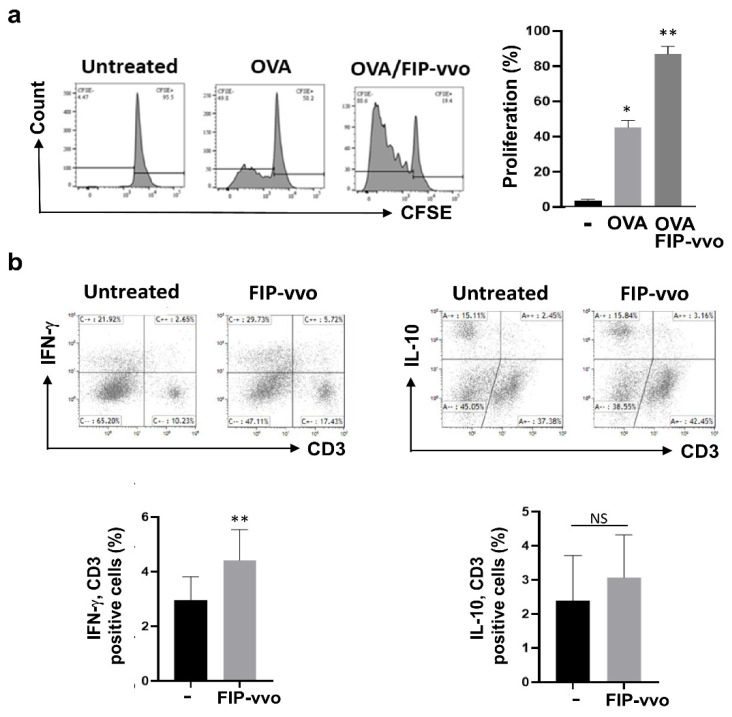
FIP-vvo-stimulated DCs prime T cell and Th1 skewing. (**a**) Mouse CD4-positive T cells are harvested from OT-II mice and labeled with CFSE. FIP-vvo is added or not added to the ovalbumin (OVA) peptide (2 μg/mL)-treated DCs for 3 h. These DCs are cocultured with T cells at 1:10 (DCs: T cells). CFSE dilution analysis by flow cytometry is examined to evaluate the proliferating ratio of T cells. Representative data and quantification are shown (mean ± SD, * *p* < 0.05, ** *p* < 0.01, control vs. DCs, *t*-test, *n* = 3 mice/group). (**b**) After DC–T cell coculture, cells are stimulated with PMA and ionomycin in the presence of GolgiPlug. The intracellular IFN-γ and IL-10 levels in CD3 T cells are detected by flow cytometry. Representative data and quantification are shown (mean ± SD; NS, no significance; * *p* < 0.05; ** *p* < 0.01; FIP-vvo vs. untreated; *t*-test; *n* = 3 mice/group).

The upregulation of costimulatory molecules and the secretion of cytokines lead DCs to promote naïve T cell activation and differentiation. To further examine whether FIP-vvo-treated DCs are able to activate naïve T cells, the antigen-specific T cell proliferation assay in vitro was performed [15,31]. CD4-positive T cells were isolated from OT-II transgenic mice, labeled with CFSE, and then cocultured with OVA_323–339_ peptide-pulsed and FIP-vvo-treated DCs for 72 h. T cell proliferation was determined by a CFSE dilution assay. As shown in Figure 4a, FIP-vvo-treated DCs showed a higher level of OVA-specific T cell proliferation than that of untreated DCs. Furthermore, the DC-produced IL-12 is able to prime T helper cell type 1 (Th1) differentiation by DC maturation [20]. To further characterize T cell subtypes that are induced by FIP-vvo-treated DCs, the IFN-γ (Th1) and IL-10 (regulatory T cells) levels were measured. FIP-vvo-treated DCs induce higher IFN-γ production, but not IL-10, compared to the untreated DCs (Figure 4b). The data demonstrated that DC maturation and activation enhanced by FIP-vvo increases the ability to activate the antigen-specific T cells and Th1 response in vitro.

### 3.4. FIP-vvo Increases DC Migration and Facilitates Specific T cell Responses

The mature DCs move to draining lymph nodes (LNs) for T cell priming. Upregulation of CCR7, a chemotactic receptor, has been found to guide DC trafficking to LNs and T cell homing [33]. 

**Figure 5:**
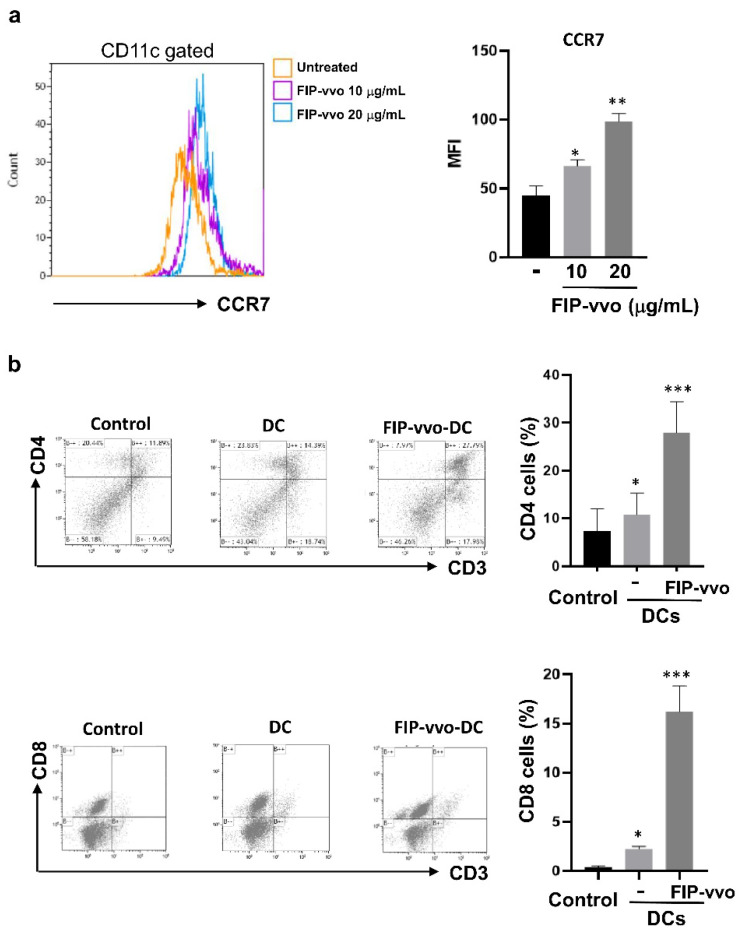
FIP-vvo treatment enhances DC migration and DC-primed T cell homing. (**a**) DCs are treated with medium (untreated/-) or FIP-vvo (10 and 20 μg/mL), and the CCR7 mean fluorescence intensity (MFI) levels of the DCs are determined by flow cytometry. Results are presented as the mean ± SD (mean ± SD, * *p* < 0.05, ** *p* < 0.01, FIP-vvo vs. untreated, *t*-test, *n* = 3 mice/group). (**b**) C57BL/6 mice are inoculated with OVA mixed with incomplete Freund’s adjuvant (IFA) alone or IFA and FIP-vvo (10 μg) via footpad injection. After 10 days, total cells are harvested from draining lymph nodes and cultured with OVA for 3 days. T cell populations were analyzed by flow cytometry. Representative data and quantification are shown. Results are presented as the mean ± SD. The comparison is between T cells from control mice and those from FIP-vvo-treated mice. * *p* < 0.05; *** *p* < 0.001. All data are representative of three independent experiments (mean ± SD, * *p* < 0.05, ** *p* < 0.01, *** *p* < 0.005, control vs. DCs, *t*-test, *n* = 3 mice/group).

Therefore, the role of FIP-vvo in the CCR7 expression level of DCs was also addressed. The current results showed that the CCR7 expression level in FIP-vvo-treated DCs was higher than that in the untreated DCs (Figure 5a). Furthermore, to analyze the effect of FIP-vvo on DC-induced T cell activation in vivo, C57BL/6 mice were immunized with OVA/IFA without (control) or with FIP-vvo via footpad injection. After 10 days, total cells from the draining lymph nodes were harvested for T cell population analysis. The FIP-vvo significantly facilitated CD4 and CD8 T cell accumulations (Figure 5b). These data support that the treatment of FIP-vvo could help DCs to elevate T cell priming and activation.

## 4. Discussion

In this study, we demonstrated that FIP-vvo from *V. volvacea* (paddy straw mushroom) promotes mouse BMDC maturation and enhances the pro-inflammatory cytokine production by BMDCs. FIP-vvo also increases BMDC-induced T cell activation in vitro. Furthermore, FIP-vvo upregulates the ability of BMDC migration and facilitates with the antigen-specific T cell response and Th1 polarization. To our knowledge, this is the first report to characterize the role of FIP-vvo in DC function and participation in the regulation of the immune response.

Several kinds of immunomodulatory molecules have been found in *V. volvacea*, such as FIPs, lectins, terpenoids, and polysaccharides. Some substances are isolated from fruiting bodies and mycelia and promote cytokine and chemokine productions. For example, the alpha-glucan from fruiting bodies of *V. volvacea* has been found to induce the expression of TNF-α, IL-6, and IL-1β in RAW264.7 macrophages [17]. In addition, lectins isolated from *V. volvacea* have been demonstrated to induce the transcriptional expression of IL-2 and IFN-γ in mouse splenocytes [34] and T lymphocytes [3]. Our data show that FIP-vvo induces the secretions of TNF-α, IL-2, IL-6, and IL-12 p70 in mouse DCs. Thus, we add one new immunomodulator in *V. volvacea.*

There are now more than 38 types of FIPs [1]. Most FIPs belong to Fve-type FIPs, such as FIP-vvo, Ling-Zhi 8 (LZ-8) from *Ganoderma lucidum* [35], FIP-gsi from *Ganoderma sinense* [36], and FIP-fve from *Flammulina velutipes* [37]. A lot of studies indicate that Fve-type FIPs can regulate innate and adaptive immunity. In contrast, the other four subgroups of FIPs mainly modulate innate immune responses, for example, macrophage activity [5]. In fact, Fve-type FIPs from different fungus species have various abilities of immune regulation, and they even share structure homology. For instance, all LZ-8, FIP-fve, and FIP-vvo can stimulate the proliferation of mouse splenocytes and human peripheral blood mononuclear cells; however, LZ-8 has the strongest effect in activating the immune response [11]. Indeed, our previous studies have demonstrated that LZ-8 is a promising adjuvant to enhance the efficacy of DNA vaccines [14,15]. Whether FIP-vvo can be used as a potential adjuvant for vaccination is not known. Similarly, FIP-fve efficiently induces T cell responses, especially Th1 responses [4]. Hsu et al. reported that FIP-vvo not only enhances Th1 responses (IL-2, TNF-α, and IFN-γ) but also induces Th2 responses (IL-4) [6]. We need more studies to understand the mechanism of FIP-vvo in activating T cells.

In addition to T cell activation, Fve-type FIPs can also activate antigen-presenting cells. LZ-8 enhances the expression of MHCII, CD80, CD86, and CD83 on DCs [8,14,15] and MHCII on macrophages [38]. FIP-fve promotes the expression of CD80, MHCI, and MHCII on peripheral blood mononuclear cells [39]. Here, we found that FIP-vvo efficiently enhances MHCII expression but not CD80 and CD86 on DCs. Therefore, the immunomodulatory activity of FIP-vvo may be lower than that of FIP-fve or LZ-8 in inducing DC maturation. Nevertheless, FIP-vvo, similar to other Fve-type FIPs, may still be used as an immunomodulator in the health food industry.

FIPs are usually present in small amounts in mushrooms. The low yield of FIPs is the main limitation for research and application. The production of recombinant FIP through gene cloning technology has greatly improved the quantity and quality of FIP [5]. In this study, the recombinant FIP-vvo proteins can enhance DC maturation and function the same as the natural FIP-vvo. Furthermore, recent studies have demonstrated that FIP-SJ75, reconstituted FIP from *G. lucidum, F. velutipes*, *and V. volvacea*, has increased immunomodulatory function on RAW264.7 macrophages [40]. Therefore, it is worthwhile to keep investigating and optimizing these FIPs.

In summary, we showed that FIP-vvo promotes mouse BMDC maturation and enhances the pro-inflammatory cytokine production by DCs. FIP-vvo increases DC-induced T cell activation and facilitates Th1 polarization in vitro and in vivo. Further work is required to figure out the mechanism of FIP-vvo that enhances DC maturation and function. Thus, FIP-vvo may have benefits as an immunomodulatory agent for human health.

## 5. Conclusions

In this study, we reported for the first time that FIP-vvo from *V. volvacea* promotes the activation and function of DCs and facilitates DC-induced T cell responses. Our findings provide evidence that FIP-vvo may potentially be developed as a functional supplement in the diet to benefit human health.

## Data Availability

Not applicable.

## References

[1] Lu H., Lou H., Hu J., Liu Z., Chen Q. (2020). Macrofungi: A review of cultivation strategies, bioactivity, and application of mushrooms. Compr. Rev. Food Sci. Food Saf..

[2] Bao D., Gong M., Zheng H., Chen M., Zhang L., Wang H., Jiang J., Wu L., Zhu Y., Zhu G. (2013). Sequencing and comparative analysis of the straw mushroom (Volvariella volvacea) genome. PLoS ONE.

[3] Sze S.C., Ho J.C., Liu W.K. (2004). Volvariella volvacea lectin activates mouse T lymphocytes by a calcium dependent pathway. J. Cell Biochem..

[4] Zhao S., Gao Q., Rong C., Wang S., Zhao Z., Liu Y., Xu J. (2020). Immunomodulatory Effects of Edible and Medicinal Mushrooms and Their Bioactive Immunoregulatory Products. J. Fungi.

[5] Liu Y., Bastiaan-Net S., Wichers H.J. (2020). Current Understanding of the Structure and Function of Fungal Immunomodulatory Proteins. Front. Nutr..

[6] Hsu H.C., Hsu C.I., Lin R.H., Kao C.L., Lin J.Y. (1997). Fip-vvo, a new fungal immunomodulatory protein isolated from Volvariella volvacea. Biochem. J..

[7] Hsieh K.Y., Hsu C.I., Lin J.Y., Tsai C.C., Lin R.H. (2003). Oral administration of an edible-mushroom-derived protein inhibits the development of food-allergic reactions in mice. Clin. Exp. Allergy.

[8] Lin Y.L., Liang Y.C., Tseng Y.S., Huang H.Y., Chou S.Y., Hseu R.S., Huang C.T., Chiang B.L. (2009). An immunomodulatory protein, Ling Zhi-8, induced activation and maturation of human monocyte-derived dendritic cells by the NF-kappaB and MAPK pathways. J. Leukoc. Biol..

[9] Hachimura S., Totsuka M., Hosono A. (2018). Immunomodulation by food: Impact on gut immunity and immune cell function. Biosci. Biotechnol. Biochem..

[10] Chu P.Y., Sun H.L., Ko J.L., Ku M.S., Lin L.J., Lee Y.T., Liao P.F., Pan H.H., Lu H.L., Lue K.H. (2017). Oral fungal immunomodulatory protein-Flammulina velutipes has influence on pulmonary inflammatory process and potential treatment for allergic airway disease: A mouse model. J. Microbiol. Immunol. Infect..

[11] Ejike U.C., Chan C.J., Okechukwu P.N., Lim R.L.H. (2020). New advances and potentials of fungal immunomodulatory proteins for therapeutic purposes. Crit. Rev. Biotechnol..

[12] Lee M.F., Chiang C.H., Lin S.J., Song P.P., Liu H.C., Wu T.J., Lin W.W. (2020). Recombinant Lactococcus lactis Expressing Ling Zhi 8 Protein Ameliorates Nonalcoholic Fatty Liver and Early Atherogenesis in Cholesterol-Fed Rabbits. BioMed Res. Int..

[13] Chen Y.H., Shin J.Y., Wei H.M., Lin C.C., Yu L.C.H., Liao W.T., Chen D.C., Chu C.L. (2021). Prevention of dextran sulfate sodium-induced mouse colitis by the fungal protein Ling Zhi-8 via promoting the barrier function of intestinal epithelial cells. Food Funct..

[14] Chu C.L., Chen Dz C., Lin C.C. (2011). A novel adjuvant Ling Zhi-8 for cancer DNA vaccines. Hum. Vaccinces.

[15] Lin C.C., Yu Y.L., Shih C.C., Liu K.J., Ou K.L., Hong L.Z., Chen J.D., Chu C.L. (2011). A novel adjuvant Ling Zhi-8 enhances the efficacy of DNA cancer vaccine by activating dendritic cells. Cancer Immunol. Immunother..

[16] Wu J.R., Hu C.T., You R.I., Ma P.L., Pan S.M., Lee M.C., Wu W.S. (2015). Preclinical trials for prevention of tumor progression of hepatocellular carcinoma by LZ-8 targeting c-Met dependent and independent pathways. PLoS ONE.

[17] Cui F., Jiang L., Qian L., Sun W., Tao T., Zan X., Yang Y., Wu D., Zhao X. (2020). A macromolecular alpha-glucan from fruiting bodies of Volvariella volvacea activating RAW264. 7 macrophages through MAPKs pathway. Carbohydr. Polym..

[18] Chen P., Liu X., Sun Y., Zhou P., Wang Y., Zhang Y. (2016). Dendritic cell targeted vaccines: Recent progresses and challenges. Hum. Vaccinces Immunother..

[19] Said A., Weindl G. (2015). Regulation of Dendritic Cell Function in Inflammation. J. Immunol. Res..

[20] Langenkamp A., Messi M., Lanzavecchia A., Sallusto F. (2000). Kinetics of dendritic cell activation: Impact on priming of TH1, TH2 and nonpolarized T cells. Nat. Immunol..

[21] Dalod M., Chelbi R., Malissen B., Lawrence T. (2014). Dendritic cell maturation: Functional specialization through signaling specificity and transcriptional programming. EMBO J..

[22] Mildner A., Jung S. (2014). Development and function of dendritic cell subsets. Immunity.

[23] Sayour E.J., Sanchez-Perez L., Flores C., Mitchell D.A. (2015). Bridging infectious disease vaccines with cancer immunotherapy: A role for targeted RNA based immunotherapeutics. J. Immunother. Cancer.

[24] Seya T., Takeda Y., Takashima K., Yoshida S., Azuma M., Matsumoto M. (2018). Adjuvant immunotherapy for cancer: Both dendritic cell-priming and check-point inhibitor blockade are required for immunotherapy. Proc. Jpn. Acad. Ser. B Phys. Biol. Sci..

[25] Vermaelen K. (2019). Vaccine Strategies to Improve Anti-cancer Cellular Immune Responses. Front. Immunol..

[26] You R.I., Lee Y.P., Su T.Y., Lin C.C., Chen C.S., Chu C.L. (2019). A Benzenoid 4,7-Dimethoxy-5-Methyl-L, 3-Benzodioxole from Antrodia cinnamomea Attenuates Dendritic Cell-Mediated Th2 Allergic Responses. Am. J. Chin. Med..

[27] Bottcher J.P., Reis E.S.C. (2018). The Role of Type 1 Conventional Dendritic Cells in Cancer Immunity. Trends Cancer.

[28] Pan Y.G., Yu Y.L., Lin C.C., Lanier L.L., Chu C.L. (2017). FcepsilonRI gamma-Chain Negatively Modulates Dectin-1 Responses in Dendritic Cells. Front. Immunol..

[29] Lin M.K., Yu Y.L., Chen K.C., Chang W.T., Lee M.S., Yang M.J., Cheng H.C., Liu C.H., Chen Dz C., Chu C.L. (2011). Kaempferol from Semen cuscutae attenuates the immune function of dendritic cells. Immunobiology.

[30] Huang R.-Y., Yu Y.-L., Cheng W.-C., OuYang C.-N., Fu E., Chu C.-L. (2010). Immunosuppressive Effect of Quercetin on Dendritic Cell Activation and Function. J. Immunol..

[31] Chu C.L., Lowell C.A. (2005). The Lyn tyrosine kinase differentially regulates dendritic cell generation and maturation. J. Immunol..

[32] Granucci F., Zanoni I., Feau S., Ricciardi-Castagnoli P. (2003). Dendritic cell regulation of immune responses: A new role for interleukin 2 at the intersection of innate and adaptive immunity. EMBO J..

[33] Braun A., Worbs T., Moschovakis G.L., Halle S., Hoffmann K., Bolter J., Munk A., Forster R. (2011). Afferent lymph-derived T cells and DCs use different chemokine receptor CCR7-dependent routes for entry into the lymph node and intranodal migration. Nat. Immunol..

[34] She Q.-B., Ng T.-B., Liu W.-K. (1998). A Novel Lectin with Potent Immunomodulatory Activity Isolated from Both Fruiting Bodies and Cultured Mycelia of the Edible MushroomVolvariella volvacea. Biochem. Biophys. Res. Commun..

[35] Tanaka S., Ko K., Kino K., Tsuchiya K., Yamashita A., Murasugi A., Sakuma S., Tsunoo H. (1989). Complete amino acid sequence of an immunomodulatory protein, ling zhi-8 (LZ-8). An immunomodulator from a fungus, Ganoderma lucidium, having similarity to immunoglobulin variable regions. J. Biol. Chem..

[36] Zhou X.-W., Xie M., Hong F., Li Q.-Z. (2009). Genomic Cloning and Characterization of a FIP-gsi Gene Encoding a Fungal Immunomodulatory Protein from *Ganoderma sinense* Zhao et al. (Aphyllophoromycetideae). Int. J. Med. Mushrooms.

[37] Ko J.L., Hsu C.I., Lin R.H., Kao C.L., Lin J.Y. (1995). A new fungal immunomodulatory protein, FIP-fve isolated from the edible mushroom, Flammulina velutipes and its complete amino acid sequence. Eur. J. Biochem..

[38] Yeh C.H., Chen H.C., Yang J.J., Chuang W.I., Sheu F. (2010). Polysaccharides PS-G and protein LZ-8 from Reishi (Ganoderma lucidum) exhibit diverse functions in regulating murine macrophages and T lymphocytes. J. Agric. Food Chem..

[39] Chang H.H., Hsieh K.Y., Yeh C.H., Tu Y.P., Sheu F. (2010). Oral administration of an Enoki mushroom protein FVE activates innate and adaptive immunity and induces anti-tumor activity against murine hepatocellular carcinoma. Int. Immunopharmacol..

[40] Shao K.-D., Mao P.-W., Li Q.-Z., Li L.-D.-J., Wang Y.-l., Zhou X.-W. (2019). Characterization of a novel fungal immunomodulatory protein, FIP-SJ75 shuffled from Ganoderma lucidum, Flammulina velutipes and Volvariella volvacea. Food Agric. Immunol..

